# Studies of Scattering, Reflectivity, and Transmitivity in WBAN Channel: Feasibility of Using UWB

**DOI:** 10.3390/s100605503

**Published:** 2010-06-02

**Authors:** Md. Humaun Kabir, Kazi Ashrafuzzaman, M. Sanaullah Chowdhury, Kyung Sup Kwak

**Affiliations:** Graduate School of IT & Telecommunication Engineering, Inha University, Yonghyun-Dong 253, Nam-Gu, Incheon 402-751, Korea; E-Mails: hakim2021@yahoo.com (M.H.K); ashraf@cu.ac.bd (K.A.); sana1691@yahoo.com (M.S.C.)

**Keywords:** reflection, transmission, scattering, propagator, attenuation

## Abstract

The Wireless Personal Area Network (WPAN) is one of the fledging paradigms that the next generation of wireless systems is sprouting towards. Among them, a more specific category is the Wireless Body Area Network (WBAN) used for health monitoring. On the other hand, Ultra-Wideband (UWB) comes with a number of desirable features at the physical layer for wireless communications. One big challenge in adoption of UWB in WBAN is the fact that signals get attenuated exponentially. Due to the intrinsic structural complexity in human body, electromagnetic waves show a profound variation during propagation through it. The reflection and transmission coefficients of human body are highly dependent upon the dielectric constants as well as upon the frequency. The difference in structural materials such as fat, muscles and blood essentially makes electromagnetic wave attenuation to be different along the way. Thus, a complete characterization of body channel is a challenging task. The connection between attenuation and frequency of the signal makes the investigation of UWB in WBAN an interesting proposition. In this paper, we study analytically the impact of body channels on electromagnetic signal propagation with reference to UWB. In the process, scattering, reflectivity and transmitivity have been addressed with analysis of approximate layer-wise modeling, and with numerical depictions. Pulses with Gaussian profile have been employed in our analysis. It shows that, under reasonable practical approximations, the human body channel can be modeled in layers so as to have the effects of total reflections or total transmissions in certain frequency bands. This could help decide such design issues as antenna characteristics of implant devices for WBAN employing UWB.

## 1. Introduction

Wireless Body Area Networks (WBANs) have attracted interest in recent years because of a number of promising applications—specifically, in the field of health monitoring. Like everyday attire, in a WBAN, several small nodes are placed directly in, on or around the human body. Since WBAN nodes acquire their power from rechargeable batteries or by energy harvesting, it is essential that they be extremely energy-efficient [1]. Above and beyond the energy efficiency, the nodes are meant to be of low complexity to keep costs down, among other things. On the other hand, Ultra-Wideband (UWB) communication is a transmission technology that comes with such promises as low-power consumption [2], interference robustness [3], high local capacity [4], and less complex hardware, most of which are highly desirable for WBANs [5]. One key concern in this regard is about signal attenuation which occurs exponentially with frequency. This leads to the need to study electromagnetic propagation across human body as medium, with consideration for UWB signal as it relates to communication system parameters for implant devices. Particularly, Impulse-Radio (IR) [6] transmission appears to be well suited to reduce complexity, since major parts of narrowband communication systems such as mixers, RF (Radio Frequency) oscillators, or Phase-Locked Loops (PLLs) can be omitted in IR systems [7]. In order to accomplish the requirements mentioned above as they relate to energy efficiency and complexity reduction, the distinct behavior of the propagation channel has to be taken into account. For WBANs, this has to do with identification of the effects of propagation on or around the body. Unlike conventional wireless channels, a human body is rather complex in structure. Electromagnetic waves show a profound variation during propagation through human body as the reflection and transmission coefficients are highly dependent upon the dielectric constants [10], in addition to the frequency. Therefore, it is a challenging task to make a complete characterization of the human body channel. Due to the differences in structural materials such as fat, muscles, blood etc., electromagnetic wave attenuation is different across the different parts of the body. The higher the frequency, the more attenuation takes place, which limits the use of high frequency or UWB in WBAN. In this manuscript, we analyze the impact of the body channel on the signals in different frequency bands. Scattering, reflectivity, and transmitivity have been studied with analysis of approximate layer-wise modeling, and with relevant numerical rendering. Pulses, having Gaussian profile, have been used in our analysis. We illustrate that the body channel can be mathematically modeled as composed of layers, with total reflection and total transmission of the signal in certain frequency bands, so as to approximate the propagation effect. The rest of this paper is organized as follows: the whole mathematical model for wave propagation in biological media, scattering, reflection, and anti-reflection by single layer is given in Section 2; Section 3 describes numerical results and concluding remarks are given in Section 4.

## 2. Mathematical Model

### 2.1. Wave Propagation through Biological Media

From the appendix A, if the incident wave is a linearly polarized uniform plane wave travelling along the z-direction, then, for ***E*** and ***H***, Equation (A.9) is of the form:

(1) $$\vec{E}=E_{i}e^{-\alpha z}e^{j\left(\omega t-\beta z\right)}i_{x}$$

(2) $$\vec{H}=H_{i}e^{-\alpha z}e^{j\left(\omega t-\beta z\right)}i_{y}$$

where *E_i_* = *ηH_i_*

The intrinsic impedance of biological material *η* is given by:

(3) $$\eta =\sqrt{\frac{\mu }{\varepsilon }}\left[1-0.378{\left(\frac{\varepsilon^{\prime \prime }}{\varepsilon^{\prime }}\right)}^{2}+j0.5\left(\frac{\varepsilon^{\prime \prime }}{\varepsilon^{\prime }}\right)\right]$$

The Pointing Vector, that is, the power flowing per unit area of cross section (W/m^2^), gives the power density associated with an EM wave:

(4) $${\vec{P}}_{i}={\vec{E}}_{i}\times {\vec{H}}_{i}$$

For a uniform plane wave, time-average power flow is given by:

(5) $$P_{i}=\frac{{\left|E_{i}\right|}^{2}}{2\eta }=\frac{1}{2}\eta {\left|H_{i}\right|}^{2}$$

The permittivity and frequency may also determine how far the EM wave penetrates into the body. The term depth of penetration (*D_p_*) usually quantifies this. For objects with homogeneous properties and with RFR incident at right angles to the surface, depth of penetration is defined as the distance at which the power density is decreased by absorption to about 0.13534 of the body’s surface value. However, the magnitude of the electric and the magnetic field reduces by a factor of 0.36788. Depth of penetration is defined as:

(6) $$D_{p}=\frac{1}{\alpha }$$

where *α* is the attenuation constant of the material in nepers per meter.

### 2.2. Scattering, Reflection, and Anti-Reflection

Sensor nodes find a human body, when they are placed, to be layered media. Fat, muscles *etc.* are such independent layers. Signals from wireless sensors in human body experience scattering, reflection, and diffraction by those layers. In Appendix B, we started with acoustic wave for mathematical formulation and then used these formulae for electromagnetic wave. We have established the basic equations for the wave propagation through the layered media. Now we will use those mathematical equations for calculating some wave properties such as scattering, reflection, and transmission while passing through layered media.

#### 2.2.1. Scattering by a Single Interface

In this section we consider the case in which two homogeneous half-spaces are separated by an interface at *z =* 0 (Figure 3):

$$\rho (z)=\{\begin{array}{c} \rho_{0}\;\mathrm{if}\;z<0\\ \rho_{1}\;\mathrm{if}\;z>0 \end{array}\;K(z)=\{\begin{array}{c} K_{0}\;\mathrm{if}\;z<0\\ K_{1}\;\mathrm{if}\;z>0 \end{array}$$

The goal of this section is to analyze the scattering problem in terms of right- and left-going modes (Appendix B).

We introduce the local velocities $c_{j}=\sqrt{K_{j}/\rho_{j}}$ and impedances $\zeta_{j}=\sqrt{K_{j}\;\rho_{j}}$ and the right- and left-going modes defined by:

(7) $$z<0:\{\begin{array}{c} A_{0}\;(t,z)=\zeta_{0}^{-1/2}p(t,z)+\zeta_{0}^{1/2}u(t,z)\\ B_{0}\;(t,z)=-\zeta_{0}^{-1/2}p(t,z)+\zeta_{0}^{1/2}u(t,z) \end{array}$$

(8) $$z>0:\{\begin{array}{c} A_{1}\;(t,z)=\zeta_{1}^{-1/2}p(t,z)+\zeta_{1}^{1/2}u(t,z)\\ B_{1}\;(t,z)=-\zeta_{1}^{-1/2}p(t,z)+\zeta_{1}^{1/2}u(t,z) \end{array}$$

For *j =* 0, 1, the pairs (*A_j_*, *B_j_*) satisfy the following system in their respective half-spaces:

(9) $$\frac{\partial }{\partial z}\left[\begin{array}{c} A_{j}\\ B_{j} \end{array}\right]=\frac{1}{c_{j}}\left[\begin{array}{cc} -1 & 0\\ 0 & 1 \end{array}\right]\frac{\partial }{\partial t}\left[\begin{array}{c} A_{j}\\ B_{j} \end{array}\right]$$

which means that *A_j_* (*t*, *z*) is a function of *t* − *z*/*c_j_* only, and *B_j_* (*t*, *z*) is a function of *t* + *z*/*c_j_* only.

We assume that a right-going wave with the time profile *f* is incoming from the left and is partly reflected by the interface. We also assume a radiation condition in the right half-space so that no wave is coming from the right. Assume that *f* is completely supported in (0, ∞). We next introduce two ways to define proper boundary conditions:

(I) We can consider an initial value problem with initial conditions given at some time *t*_0_ < 0 by:

(10) $$u(t=t_{0},z)=\frac{1}{2\zeta_{0}^{1/2}}f(t_{0}-z/c_{0}),\;p(t=t_{0},z)=\frac{\zeta_{0}^{1/2}}{2}f(t_{0}-z/c_{0})$$

As shown in the Appendix B, these initial conditions generate a pure right-going wave whose support at *t = t_0_* is in the interval *z* ∈ (−∞, *c*_0_*t*_0_), which lies in the left half-space.

(II) We can consider a point source located at some point *z*_0_ < 0 and generating a forcing term of the from:

(11) $$F(t,z)=\zeta_{0}^{1/2}f(t-z_{0}/c_{0})\delta (z-z_{0})$$

As seen in the Appendix B, this point source generates two waves. The left-going wave is propagating into the negative *z*-direction and will never interact with the interface, so we will ignore it. The right-going wave first propagates in the homogeneous left half-space and it eventually interacts with the interface *z =* 0.

In terms of the right- and left-going waves, these two formulations give the same descriptions. We have *A*_0_ (*t*, *z*) = *f*(*t* − *z*/*c*_0_) *for z* < 0, and B_1_(t, z) = 0 for *z* > 0, and consequently, at the interface *z =* 0:

(12) $$A_{0}(t,0)=f(t),\;\;B_{1}(t,0)=0$$

Note that the delays introduced in the initial conditions (10) and in the forcing term (11) have been chosen so that the boundary conditions (12) have a very simple form.

The pairs (*A*_0_, *B*_0_) and (*A*_1_, *B*_1_) are coupled by the jump conditions at *z =* 0 corresponding to the continuity of the velocity and pressure fields:

$$\begin{array}{l} u(t,0)=\zeta_{0}^{-1/2}\left(\frac{A_{0}(t,0)+B_{0}(t,0)}{2}\right)=\zeta_{1}^{-1/2}\left(\frac{A_{1}(t,0)+B_{1}(t,0)}{2}\right)\\ p(t,0)=\zeta_{0}^{1/2}\left(\frac{A_{0}(t,0)-B_{0}(t,0)}{2}\right)=\zeta_{1}^{1/2}\left(\frac{A_{1}(t,0)-B_{1}(t,0)}{2}\right) \end{array}$$

which gives:

(13) $$\left[\begin{array}{c} A_{1}(t,0)\\ B_{1}(t,0) \end{array}\right]=J\left[\begin{array}{c} A_{0}(t,0)\\ B_{0}(t,0) \end{array}\right],\;\;\;\;J=\left[\begin{array}{cc} r^{\left(+\right)} & r^{\left(-\right)}\\ r^{\left(-\right)} & r^{\left(+\right)} \end{array}\right]$$

with $r^{\left(\pm \right)}=\frac{1}{2}\left(\sqrt{\zeta_{1}/\zeta_{0}}\pm \sqrt{\zeta_{0}/\zeta_{1}}\right)$. Note that (*r*^(+)^)^2^ – (*r*^(−)^)^2^ = 1. The matrix ***J*** can be interpreted as a propagator, since it “propagates” the right- and left-going modes from the left side of the interface to the right side. Such a propagator matrix will be called interface propagator in the following.

Taking into account the boundary conditions (12) yields:

$$\left[\begin{array}{c} A_{1}(t,0)\\ 0 \end{array}\right]=J\left[\begin{array}{c} f(t)\\ B_{0}(t,0) \end{array}\right]$$

and solving this equation gives:

$$B_{0}(t,0)=𝒭\;f(t),\;A_{1}(t,0)=𝒯\;f(t)$$

where 𝒭 and 𝒯 are the reflection and transmission coefficients of the interface:

$$𝒭=-\frac{r^{\left(-\right)}}{r^{\left(+\right)}}=\frac{\zeta_{0}-\zeta_{1}}{\zeta_{0}+\zeta_{1}},\;𝒯=\frac{1}{r^{\left(+\right)}}=\frac{2\sqrt{\zeta_{0}\zeta_{1}}}{\zeta_{0}+\zeta_{1}}$$

These coefficients satisfy the energy-conservation relation:

$${𝒭}^{2}+{𝒯}^{2}=1$$

meaning that the sum of the energies of the reflected and transmitted waves is equal to the energy of the incoming waves. Finally, the complete solution for *z* < 0 in terms of the right- and left-going modes is:

$$A_{0}(t,z)=f(t-z/c_{0}),\;B_{0}(t,z)=𝒭f(t+z/c_{0})$$

and for *z* > 0:

$$A_{1}(t,z)=𝒯\;f(t-z/c_{1}),\;B_{1}(t,z)=0$$

Using (7–8) we can obtain the pressure and velocity fields (Figure 4).

#### 2.2.2. Single-Layer Case: Scattering

In this section, we consider the case of a homogeneous slab with thickness L embedded between two homogeneous half-spaces (Figure 5). Three regions can be described as follows:

$$\rho (z)=\{\begin{array}{c} \rho_{0}\;\mathrm{if}\;z<0,\\ \rho_{1}\;\mathrm{if}\;z\in [0,L],\\ \rho_{2}\;\mathrm{if}\;z<0, \end{array}\;\;K(z)=\{\begin{array}{c} K_{0}\;\mathrm{if}\;z<0\\ K_{1}\;\mathrm{if}\;z\in [0,L]\\ K_{2}\;\mathrm{if}\;z<0 \end{array}$$

We introduce the local velocities $c_{j}=\sqrt{K_{j}/\rho_{j}}$ and impedances $\zeta_{j}=\sqrt{K_{j}\;\rho_{j}}$ and the local right- and left-going modes defined by:

$$A_{j}\;(t,z)=\zeta_{j}^{-1/2}p\;(t,z)+\zeta_{j}^{1/2}u\;(t,z),B_{j}\;(t,z)=-\zeta_{j}^{-1/2}p\;(t,z)+\zeta_{j}^{1/2}u\;(t,z)$$

with *j* = 0 for *z* < 0, *j* = 1 for *z* ∈ [0, *L*], and *j* = 2 for *z* = *L*. The boundary conditions correspond to an impinging pulse at the interface *z* = 0 and a radiation condition at *z* = *L*_2_:

$$A_{0}(t,\;0)=f(t),B_{2}(t,L)=0$$

The propagation equations (9) in each homogeneous region show that *A_j_* is a function of *t* − *z* / *c_j_* only and *B_j_* is a function of *t* + *z* / *c_j_* only. The waves inside the slab [0, L] are therefore of the form:

$$A_{1}(t,z)=a_{1}(t-z/c_{1}),B_{1}(t,z)=b_{1}(t+z/c_{1})$$

while the reflected wave for *z* <0 is of the form:

$$B_{0}(t,z)=b_{0}(t+z/c_{0})$$

and the transmitted wave for *z* > *L* is of the form:

$$A_{2}(t,z)=a_{2}\left(t-\frac{z-L}{c_{2}}\right)$$

We want to indentify the functions *b*_0_ and *a*_2_, which give the shapes of the reflected and transmitted waves.

#### 2.2.3. Single-Layer Case: Reflection and Transmission Coefficients

The unknown functions b_0_ and a_2_ can be obtained from the continuity conditions for the velocity and pressure at the two interfaces. At *z* = 0, we have:

$$\left[\begin{array}{c} A_{1}(t,0)\\ B_{1}(t,0) \end{array}\right]=J_{0}\;\left[\begin{array}{c} A_{0}(t,0)\\ B_{0}(t,0) \end{array}\right],\;J_{0}=\left[\begin{array}{cc} r_{0}^{\left(+\right)} & r_{0}^{\left(-\right)}\\ r_{0}^{\left(-\right)} & r_{0}^{\left(+\right)} \end{array}\right]$$

with $r_{0}^{\left(\pm \right)}=\frac{1}{2}(\sqrt{\zeta_{1}/\zeta_{0}}\pm \sqrt{\zeta_{0}/\zeta_{1}})$. Similarly, at z = L:

$$\left[\begin{array}{c} A_{2}(t,L)\\ B_{2}(t,L) \end{array}\right]=J_{1}\;\left[\begin{array}{c} A_{1}(t,L)\\ B_{1}(t,L) \end{array}\right],\;J_{1}=\left[\begin{array}{cc} r_{1}^{\left(+\right)} & r_{1}^{\left(-\right)}\\ r_{1}^{\left(-\right)} & r_{1}^{\left(+\right)} \end{array}\right]$$

with $r_{1}^{\left(\pm \right)}=\frac{1}{2}(\sqrt{\zeta_{2}/\zeta_{1}}\pm \sqrt{\zeta_{1}/\zeta_{2}})$. We can write these relations in terms of the functions *a_j_*, *b_j_* as:

$$\left[\begin{array}{c} a_{1}(t)\\ b_{1}(t) \end{array}\right]=J_{0}\;\left[\begin{array}{c} f(t)\\ b_{0}(t) \end{array}\right],\;\left[\begin{array}{c} a_{2}(t)\\ 0 \end{array}\right]=J_{1}\;\left[\begin{array}{c} a_{1}(t-L\;/c_{1})\\ b_{1}(t+L\;/c_{1}) \end{array}\right]$$

which can be solved to get the reflected and transmitted waves. The situation is more complicated than in the case of a single interface, because of the time delays ±*L*/*c*_1_. A convenient and general way to handle these delays is by going to the frequency domain, so that the time shifts are replaced by phase factors. The Fourier transforms of the modes are defined by:

$${\hat{a}}_{j}\;(\omega )=\int a_{j}\;(t)e^{i\omega t}\mathrm{dt},\;{\hat{b}}_{j}\;(\omega )=\int a_{j}\;(t)e^{i\omega t}\mathrm{dt}$$

They satisfy the interface conditions:

(14) $$\left[\begin{array}{c} {\hat{a}}_{1}(\omega )\\ {\hat{b}}_{1}(\omega ) \end{array}\right]=J_{0}\;\left[\begin{array}{c} \hat{f}(\omega )\\ {\hat{b}}_{0}(\omega ) \end{array}\right],\;\left[\begin{array}{c} {\hat{a}}_{2}(\omega )\\ 0 \end{array}\right]=J_{1}\;\left[\begin{array}{c} {\hat{a}}_{1}(\omega )e^{i\frac{\omega L}{c_{1}}}\\ {\hat{b}}_{1}(\omega )e^{-i\frac{\omega L}{c_{1}}} \end{array}\right]$$

where we have used the identity:

$$\int a_{1}(t-L\;/\;c_{1})\;e^{i\omega t}\mathrm{dt}=\int a_{1}(s)\;e^{i\omega \left(s+\frac{L}{c_{1}}\right)}\mathrm{ds}={\hat{a}}_{1}(\omega )\;e^{i\frac{\omega L}{c_{1}}}$$

Introducing the frequency-dependent matrix:

$${\hat{J}}_{1}(\omega )=\left[\begin{array}{cc} r_{1}^{(+)\;\;}e^{i\frac{\omega L}{c_{1}}} & r_{1}^{(-)\;\;}e^{-i\frac{\omega L}{c_{1}}}\\ r_{1}^{(-)\;\;}e^{i\frac{\omega L}{c_{1}}} & r_{1}^{(+)\;\;}e^{-i\frac{\omega L}{c_{1}}} \end{array}\right]$$

The second equation of (14) can be written as:

(15) $$\left[\begin{array}{c} {\hat{a}}_{2}(\omega )\\ 0 \end{array}\right]={\hat{J}}_{1}(\omega )\left[\begin{array}{c} {\hat{a}}_{1}(\omega )\\ {\hat{b}}_{1}(\omega ) \end{array}\right]$$

The syplectic matrix ***Ĵ***_1_(*ω*) is a propagator in the frequency domain. It propagates the right- and left-going modes from the right side of the interface 0 to the right side of the interface 1, and it depends on the layer thickness L. Finally, combining the first equation of (14) and (15), we obtain the relation:

(16) $$\left[\begin{array}{c} {\hat{a}}_{2}(\omega )\\ 0 \end{array}\right]={\hat{K}}_{0}(\omega )\left[\begin{array}{c} \hat{f}(\omega )\\ {\hat{b}}_{0}(\omega ) \end{array}\right]$$

where the frequency-dependent syplectic matrix:

$${\hat{K}}_{0}(\omega )={\hat{J}}_{1}(\omega )J_{0}=\left[\begin{array}{cc} \hat{U}(\omega ) & \bar{\hat{V}(\omega )}\\ \hat{V}(\omega ) & \bar{\hat{U}(\omega )} \end{array}\right]$$

is the overall propagator of the slab. Equation (16) shows that K̂_0_(ω) propagates the right- and left-going modes from the left side of the interface 0 to the right side of the interface 1. We find explicitly:

$$\begin{array}{l} \hat{U}(\omega )=r_{0}^{\left(+\right)}r_{1}^{\left(+\right)}e^{i\frac{\omega L}{c_{1}}}+r_{0}^{\left(-\right)}r_{1}^{\left(-\right)}e^{-i\frac{\omega L}{c_{1}}}\\ \hat{V}(\omega )=r_{0}^{\left(+\right)}r_{1}^{\left(-\right)}e^{i\frac{\omega L}{c_{1}}}+r_{0}^{\left(-\right)}r_{1}^{\left(+\right)}e^{-i\frac{\omega L}{c_{1}}} \end{array}$$

By solving equation (16), whose unknowns are *â*_2_ (*ω*) and *b̂*_0_ (*ω*) and using the expressions of $r_{j}^{\left(\pm \right)}$, we obtain:

$${\hat{b}}_{0}(\omega )=\hat{𝒭}(\omega )\hat{f}(\omega ),\;{\hat{a}}_{2}(\omega )=\hat{𝒯}(\omega )\hat{f}(\omega )$$

where the frequency-dependent reflection and transmission coefficients are:

(17) $$\hat{𝒭}(\omega )=-\frac{\hat{V}(\omega )}{\bar{\hat{U}(\omega )}}=\frac{R_{1}e^{2i\frac{\omega L}{c_{1}}}+R_{0}}{1+R_{0}R_{1}e^{2i\frac{\omega L}{c_{1}}}}$$

(18) $$\hat{𝒯}(\omega )=\frac{1}{\bar{\hat{U}(\omega )}}=\frac{T_{0}T_{1}e^{i\frac{\omega L}{c_{1}}}}{1+R_{0}R_{1}e^{2i\frac{\omega L}{c_{1}}}}$$

using that |*Û*(*ω*)|^2^ − |*V̂*(*ω*)|^2^ = 1. Here $R_{0}=\frac{\zeta_{0}-\zeta_{1}}{\zeta_{0}+\zeta_{1}}$, $R_{1}=\frac{\zeta_{1}-\zeta_{2}}{\zeta_{1}+\zeta_{2}}$, $T_{0}=\frac{2\sqrt{\zeta_{0}\zeta_{1}}}{\zeta_{0}+\zeta_{1}}$, and $T_{1}=\frac{2\sqrt{\zeta_{1}\zeta_{2}}}{\zeta_{1}+\zeta_{2}}$ are the reflection and transmission coefficients of the two interfaces. The reflection and transmission coefficients of the layer satisfy the energy conservation relation | 𝒭̂ (*ω*)|^2^ + | 𝒯̂(*ω*)|^2^ = 1 for all *ω*, which means that the individual energies of the frequency components of the incoming pulse are preserved by the scattering process. The main qualitative difference between the scattering by a single interface and the scattering by single layer is that the reflection and transmission coefficients in the layer case are frequency-dependent. This frequency dependence originates from interference effects between the waves that are scattered back and forth by the two interfaces of the layer.

### 2.3. Filtering Property of the Layer

#### 2.3.1. Reflection

Let us consider a layer embedded between two homogeneous half-spaces that have the same material properties, *i.e.*, the situation in which *ρ*_2_ = *ρ*_0_ and *K*_2_ = *K*_0_. We then have *R*_1_ = −*R*_0_ and *T*_1_ = *T*_0_, which implies that the global reflectivity of the layer can be written as:

(19) $${\left|\hat{𝒭}(\omega )\right|}^{2}=1-\frac{1+R_{0}^{4}-2R_{0}^{2}}{1+R_{0}^{4}-2R_{0}^{2}\;\cos\left(\frac{2\omega L}{c_{1}}\right)}$$

The reflectivity is periodic with respect to the angular frequency ω with the period *ω_c_* = *πc*_1_/*L*. As a function of the angular frequency the reflectivity goes from the minimal value:

$${\left|\hat{𝒭}\right|}_{\min}^{2}=0\;\mathrm{for}\;\omega ={k\omega }_{c},k\in \mathbb{Z}$$

to the maximal value:

$${\left|\hat{𝒭}\right|}_{\max}^{2}=1-{\left(\frac{1-R_{0}^{2}}{1+R_{0}^{2}}\right)}^{2}\;\mathrm{for}\;\omega =\left(k+\frac{1}{2}\right)\omega_{c},k\in \mathbb{Z}$$

This shows that for any value of the reflection coefficient *R*_0_ of a single interface, there exist frequencies that are fully transmitted or fully reflected by the layer. If we consider the case of strong scattering $T_{0}^{2}\ll 1$, then the transmitted frequency bands have a width of the order of $\omega_{c}T_{0}^{2}$ around the fully transmitted frequencies *kω_c_*. Outside of these bands, where total reflection occurs, the typical reflectivity is large, of order $1-T_{0}^{4}/4$.

#### 2.3.2. Anti-Reflection

The total transmission phenomenon is also encountered in situations in which the two half-spaces are different. Indeed, consideration of human body part as an ideal, fully-transmitting layer is certainly beyond perfection. In a microscopic or constituent-wise sense, a human body-part, striated muscle for example, iscomposed of water (70.09%), ether-soluble extract (6.60%), crude protein (21.94%), *etc*. [35]. When one is faced with the task of modeling portions of human body as a medium for electromagnetic (signal) propagation, there are inevitable practical assumptions and approximations to be made. Thinner layers can be considered with more homogeneous characteristics, while for thicker setting with internal variability, the aggregate behavior sums up by and large. Thus a certain part of a human body like muscle or fat, when considered in macroscopic perspective, can be regarded as a continuum, and hence the idea of a planner-layered-medium assumption of human body tissues contextually holds for all practical modeling considerations. Such model could greatly affect related system design pertaining to crucial parameters (antenna and others); for instance, in addition to the frequency and bandwidth employed for the signal, the measurement of the depth of the body at which the transceiver of an implant device would function with acceptable accuracy has a lot to do with such parameters as permittivity, permeability, and impedance of the intermittent layers of body tissues.

Figure 7 shows a grossly approximated propagation system consisting of three layers: air, human body channel, and a transceiver. Admittedly, finding fully-transmitting or fully-reflecting layers in body-parts is unrealistic, but it is widely adopted practice to model systems using phantoms comprised of components having somewhat homogeneous characteristics, yet closely resembles body-parts in aggregate behavior. In some recent works ([33] and [34]) UWB antenna impedance matching has been studied in the context of biomedical implants. We assume that the two homogeneous half spaces (Figure 7) have different impedances *ζ*_0_ ≠ *ζ*_2_, then it is possible to choose the thickness L and the impedance *ζ*_1_ of the layer so that a given frequency ω will be fully transmitted from one half-space to the other one, which would not be the case in absence of such a layer. From the analysis of the reflectivity function:

(20) $${\left|\hat{R}(\omega )\right|}^{2}=1-\frac{1-R_{0}^{2}-R_{1}^{2}+R_{0}^{2}R_{1}^{2}}{1+{2R}_{0}R_{1}\;\cos\left(\frac{2\omega L}{c_{1}}\right)+R_{0}^{2}R_{1}^{2}}$$

One can show that a necessary and sufficient condition for | 𝒭̂(*ω*)|^2^ to be zero is that $R_{0}^{2}+R_{1}^{2}=-2R_{0}R_{1}\text{cos}\left(\frac{2\omega L}{c_{1}}\right)$. In the case *ζ*_0_ ≠ *ζ*_2_ this in turn enforces one to choose the impedance of the layer to be $\zeta_{1}=\sqrt{\zeta_{0}\zeta_{2}}$ (so that *R*_0_ = *R*_1_) and the thickness *L* to be chosen so that *ωL*/(π*c*_1_) is half an integer (so that cos(2 *ωL*/*c*_1_) = −1). Usually the thickness is chosen to be equal to a quarter of the wavelength, meaning *ωL*/(π*c*_1_) = ½.

### 2.4. Path Loss in the Human Body (Near Field Far Field Consideration)

When EM RF waves propagate in freespace, the power received decreases at a rate of (1/*d*)*^n^*, *n* being the coefficient of pathloss. Other kinds of losses would be fading of signals due to multipath propagation. However, for propagation of EM waves in a lossy medium like human tissue, the losses would be mainly due to absorption of power in the tissue, where it is dissipated as heat. As the tissue medium is lossy and mostly consists of water, the EM waves are attenuated considerably before they reach the receiver. *The Specific Absorption Rate* (SAR) is useful in determining the amount of power lost due to heat dissipation. SAR is defined as power absorbed per unit mass of the tissue [29]. SAR is a standard measure of how much power is absorbed in the tissue and depends upon *E*- and *H*-field strengths. By determining the average SAR over the entire mass of the tissue between the transmitter and the receiver, we are able to compute the total power lost. SAR in the near field of the transmitting antenna depends mainly on the *H*-field, whereas the SAR in the far field of the transmitting antenna depends mainly on the *E*-field. We use Maxwell’s *E*- and *H*-fields equations for lossy medium to obtain the average SAR of the medium between the transmitting and the receiving antenna in the far field and near field, respectively. WBAN applications involve wireless communications between implanted biosensor nodes inside human body.

These nodes exchange data among themselves and also with the base-station. In general, the system model consists of numerous biosensor nodes placed inside the various parts of the human body surrounded by tissues. In particular, for the development of this model, we consider only one transmitting and one receiving antenna separated by a distance *d*. An elemental short dipole (dipole length_wavelength) in a lossy human tissue medium is considered for this purpose [28] and is shown in Figure 9. A small area of tissue surrounding the antenna is considered for our analysis. Thus we can safely assume the human tissue under consideration to be a homogeneous medium with no sharp edges, no rough surfaces and having uniform electric and magnetic properties. The received power is assumed to be due only to the power from the transmitter and not from any other source. The space around the radiating antenna is divided into near field and far field regions as shown in Figure 10.

The region of space immediately surrounding the antenna is known as the near field region. The extent of the near field in the case of short dipoles is given by *d*_0_ = *λ*/2, where *λ* is the wavelength [30]. In the near field, the *E*- and *H*-field strengths vary rapidly with the distance from the antenna. The far field is the entire region beyond the near field. In the far field region, the *E*- and *H*-field exhibit a plane wave behavior. Power absorbed between the transmitting and receiving antennas can be considered as the sum of power absorbed in near field (*P_NF_*) and far field (*P_FF_*) regions. The total power absorbed between the two antennas is computed by numerical integration.

Consider an elemental oscillating electric dipole in a lossy medium of conductivity σ (S/m), permittivity *ɛ* (F/m), permeability *μ* (H/m), complex propagation constant γ, complex intrinsic impedance $\eta =\frac{\gamma }{\alpha +j\omega ɛ}$ [28] at frequency *ω*, as shown in Figure 8. The dipole consists of a short conducting wire of length *dl*, terminated in two small conductive spheres or disks. Assume that the current *I* is uniform and varies sinusoidally with time [28]. The electromagnetic field at a distance ‘*R*’ for an Hertzian dipole is derived from the vector potential *A*, given by [28]:

$$A=a_{z}\frac{\mu \mathrm{Idl}}{4\pi }\frac{e^{-\gamma R}}{R}=a_{z}A_{z}$$

where *a_z_* is the unit vector in the *z*-direction, *γ* is the propagation constant, given by *γ* = *α* + *jβ*; attenuation constant *α* and phase constant *β* is given by as [28]:

$$\begin{array}{c} \alpha =\omega \sqrt{\frac{\mu \varepsilon }{2}}{\left[\sqrt{1+{\left(\frac{\sigma }{\omega \varepsilon }\right)}^{2}}-1\right]}^{1/2}\;\;\;(\text{Neper}/m)\\ \beta =\omega \sqrt{\frac{\mu \varepsilon }{2}}{\left[\sqrt{1+{\left(\frac{\sigma }{\omega \varepsilon }\right)}^{2}}+1\right]}^{1/2}\;\;\;(\text{rad}/m) \end{array}$$

Spherical components of A (*i.e.*, *a_R_A_R_ + a_θ_A_θ_ + a_φ_A_φ_*) are given by *A_R_ = A_z_cosθ*, *A_θ_ =* −*A_z_singθ* and *A_φ_* = 0. The magnetic field intensity *H* and the electric field intensity *E* is given by [28]:

$$\begin{array}{c} H=\frac{1}{\mu }(\nabla \times A)=a_{\varphi }\frac{1}{\mu R}\left[\frac{\partial }{\partial R}({\mathrm{RA}}_{\theta })-\frac{\partial }{\partial \theta }A_{R}\right]\\ E=\frac{1}{\alpha +j\omega ɛ}(\nabla \times H)=\frac{1}{\alpha +j\omega ɛ}\left[a_{R}\frac{1}{\mathrm{Rsin}\theta }\frac{\partial }{\partial \theta }(H_{\varphi }\sin\theta )-a_{\theta }\frac{1}{R}\frac{\partial }{\partial R}({\mathrm{RH}}_{\varphi })\right] \end{array}$$

Solving the above magnetic and electric field equations for lossy medium and expressing in terms of complex impedance *η* we get:

(21) $$E_{R}=\eta \frac{2\mathrm{Idlcos}\theta }{4\pi }e^{-\gamma R}\;\left(\frac{1}{\gamma R^{3}}+\frac{1}{R^{2}}\right)$$

(22) $$E_{\theta }=\eta \frac{\mathrm{Idlsin}\theta }{4\pi }e^{-\gamma R}\;\left(\frac{1}{\gamma R^{3}}+\frac{1}{R^{2}}+\frac{\gamma }{R}\right)$$

(23) $$H_{\varphi }=\frac{\mathrm{Idlsin}\theta }{4\pi }e^{-\gamma R}\;\left(\frac{1}{R^{2}}+\frac{\gamma }{R}\right)$$

#### 2.4.1. Power Absorbed in the Near Field

The SAR in the near field is given by [31]:

$$\mathrm{SAR}=\frac{\sigma }{\rho }\frac{\mu \omega }{\rho \sqrt{\sigma^{2}+{ɛ}^{2}\omega^{2}}}{\left(1+c_{\mathrm{corr}}\;\tau \right)}^{2}H_{\mathrm{rms}}^{2}\;\mathrm{watts}/\mathrm{Kg}$$

where *ρ* is the density of the medium and *c_corr_* is the correction factor to take into account the changed reflection properties for small distances R of the antenna from the scatterer. Since we assume both the transmitting and receiving antennae are in a same homogeneous medium, the plane wave reflection coefficient *τ* is zero. By substituting *τ* = 0 and RMS value of the *H*-field, the above equation reduces to:

$$\mathrm{SAR}=\frac{\sigma }{\rho }\frac{\mu \omega }{\rho \sqrt{\sigma^{2}+{ɛ}^{2}\omega^{2}}}{\left(\frac{\mathrm{Idlsin}\theta }{4\pi }e^{-\alpha R}\;\left(\frac{1}{R^{2}}+\frac{\left|\gamma \right|}{R}\right)\right)}^{2}$$

which gives the value of SAR at a point at distance ‘R’ and angle ‘*θ*’ from the dipole. Power at infinitely small volume (*dV* = *R*^2^ *sinθ dR dθ dφ*) is:

(24) ΔP=SAR×Δmass=SAR×ρ×dV

The power absorbed in the near field of the lossy tissue can be obtained by computing the average SAR over the entire tissue mass in the near field, which is obtained by integrating Δ*P* over the entire mass in the near field region, *i.e*., from the surface of the antenna (*R* = *r*) to the end of the near-field region (*R* = *d*_0_):

$$\begin{array}{c} P_{\mathrm{NF}}=\int_{R=r}^{d_{0}}\int_{\theta =0}^{\pi }\int_{\varphi =0}^{2\pi }\Delta P\\ =\sigma \frac{\mu \omega }{\sqrt{\sigma^{2}+{ɛ}^{2}\omega^{2}}}{\left(\frac{\mathrm{Idl}}{4\pi }\right)}^{2}\int_{R=r}^{d_{0}}\int_{\theta =0}^{\pi }\int_{\varphi =0}^{2\pi }R^{2}\sin^{3}\theta \times \;e^{-2\alpha R}\left(\frac{1}{R^{4}}+\frac{{\left|\gamma \right|}^{2}}{R^{2}}+\frac{2\left|\gamma \right|}{R^{3}}\right)\mathrm{dRd}\theta d\varphi \end{array}$$

Solving by numerical integration and writing $\frac{1}{\sqrt{\sigma^{2}+{ɛ}^{2}\omega^{2}}}$ as |*η*|/|*γ*|:

(25) $$P_{\mathrm{NF}}=\sigma \mu \omega \frac{\left|\eta \right|}{\left|\gamma \right|}\frac{I^{2}{\mathrm{dl}}^{2}}{6\pi }[A+B+C]$$

where:

$$\begin{array}{l} A=e^{-2\alpha r}\left(\frac{{\left|\gamma \right|}^{2}}{2\alpha }+\frac{d_{0}-r}{{4r}^{2}}+\frac{\left|\gamma \right|(d_{0}-r)}{2r}\right)\\ B=e^{-2\alpha d_{0}}\left(\frac{-{\left|\gamma \right|}^{2}}{2\alpha }+\frac{d_{0}-r}{{4d}_{0}^{2}}+\frac{\left|\gamma \right|(d_{0}-r)}{{2d}_{0}}\right)\\ C=e^{-\alpha (d_{0}+r)}\left(\frac{2(d_{0}-r)}{{\left(d_{0}+r\right)}^{2}}+\frac{2\left|\gamma \right|(d_{0}-r)}{\left(d_{0}+r\right)}\right) \end{array}$$

The antenna dimensions depend on the wavelength of the wave in the medium given by $\lambda_{m}=\frac{2\pi }{\beta }$ [28].

#### 2.4.2. Power Absorbed in the Far Field

Neglecting $\frac{1}{R^{2}},\frac{1}{R^{3}}\ldots \;\;..$ terms from field Equations (21)–(23) for the far field, we have:

$$\begin{array}{c} E_{R}=0,E_{\theta }=\eta \frac{\mathrm{Idlsin}\theta }{4\pi }e^{-\gamma R}\;\left(\frac{\gamma }{R}\right)\\ H_{\varphi }=\frac{\mathrm{Idlsin}\theta }{4\pi }e^{-\gamma R}\left(\frac{\gamma }{R}\right) \end{array}$$

In the far field the specific absorption rate depends only on the *E_rms_* value which is given by [29]:

$$\begin{array}{c} \mathrm{SAR}=\frac{\sigma }{\rho }E_{\mathrm{rms}}^{2}\;\mathrm{watts}/\mathrm{Kg}\\ =\frac{\sigma }{\rho }{\left(\left|\eta \right|\left|\gamma \right|\frac{\mathrm{Idlsin}\theta }{4\pi R}e^{-\alpha R}\right)}^{2} \end{array}$$

The power absorbed in the infinitely small volume (*dV* = *R*^2^ *sinθ dR dθ dφ*) in the far field, at a distance R and angle *θ* from the dipole can again be obtained from (24):

$$\Delta P=\sigma {\left(\left|\eta \right|\left|\gamma \right|\frac{\mathrm{Idl}}{4\pi }\right)}^{2}\;\sin^{3}{\theta e}^{-2\alpha R}\;\mathrm{dR}\;d\theta \;d\varphi$$

The total power absorbed in the far field of the lossy tissue between the source and destination antennas can be obtained by computing the average SAR over the entire tissue mass in the far field from distance *d*_0_ to *d* (*d*_0_ is the point where the far field starts). This is obtained by integrating Δ*P* over the mass in the far field between the two antennas:

(26) $$P_{\mathrm{FF}}=\int_{R=d_{0}}^{d}\int_{\theta =0}^{\pi }\int_{\varphi =0}^{2\pi }\Delta P=\sigma {\left|\eta \right|}^{2}{\left|\gamma \right|}^{2}\frac{I^{2}{\mathrm{dl}}^{2}\mathrm{dl}}{12\pi \alpha }(e^{-2\alpha d_{0}}-e^{-2\alpha d})$$

##### Power received

The effective radiated power (ERP) is obtained by subtracting the loss in the near field (*P_NF_*) and far field (*P_FF_* between the transmitting and receiving antennas) from the transmitted power *P_T_* (*i.e*., (*P_T_* −*P_Loss_*)*G_t_*), where *P_Loss_* = *P_NF_* +*P_FF_* is obtained from (25) and (26). The power density (*P_e_*, Power per unit area) at a distance ’d’ is different in near field and far field regions:

- P_R_ in the Near Field: There is no general formula for the estimation of field strength in the near field zone [30]. Only measurements can provide a simple means of field evaluation. However, reasonable calculations can be made for antennas like dipole or monopole. When the receiving antenna is in the near field region of the transmitting antenna, the power density does not necessarily depend on the distance from the antenna, but varies rapidly with distance, and may exhibit oscillatory behavior. The magnitude of on-axis (main beam) power density varies according to the location in the near field and its maximum value is approximated by [32] *P_e_* = 16 *δP* / π*L*^2^, where *L* is the largest dimension of the antenna, *P* is *P_T_* − *P_NF_*, and *δ* is the aperture efficiency (typically 0.5–0.75) [32]. It can be approximated as *δ* = *A_e_*/*A* (*A_e_* is the effective aperture and *A* is the physical area of the antenna). The power received by the receiving antenna in the near field can be approximated by:
  $$P_{R}=P_{e}A_{e}=\frac{16\delta (P_{T}-P_{\mathrm{NF}})}{\pi L^{2}}A_{e}$$
- P_R_ in the Far Field: On the other hand when the receiving antenna is in the far field region of the transmitting antenna, the power density is dependent on the distance *d* and is given by:
  $$P_{e}=\frac{\left(P_{T}-P_{\mathrm{Loss}}\right)}{4\pi d^{2}}G_{t}$$

The power received by the receiving antenna in the far field is *P_R_* = *P_e_A_e_*, where the receiving antenna aperture *A_e_* is given by $A_{e}=\frac{\lambda^{2}}{4\pi }G_{r}$ Here, *G_t_* and *G_r_* are the gain of the transmitting and receiving antenna, respectively. Thus the received power is:

$$P_{R}=\frac{(P_{T}-P_{\mathrm{NF}}-P_{\mathrm{FF}})\lambda^{2}}{{\left(4\pi d\right)}^{2}}G_{t}G_{r}$$

and a total phase change of *e*^−*jβ*^ is involved during the propagation of the wave. Thus, PMBA can be used for calculating the propagation loss using the two Equations (25) and (26).

## 3. Numerical Results

**Figure 1.**
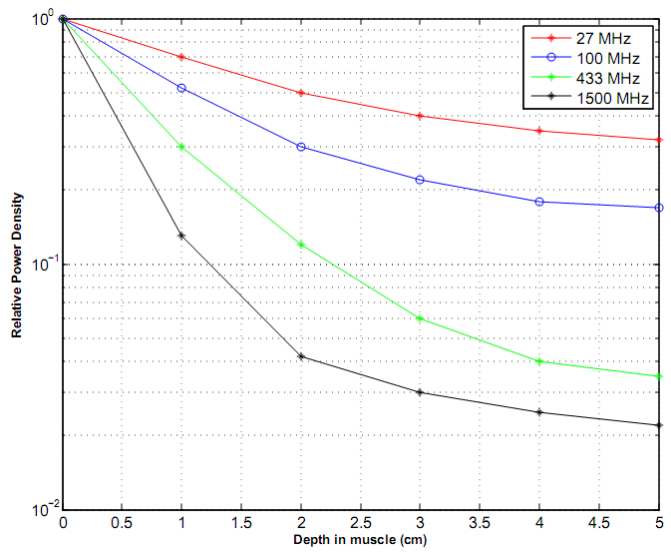
Power absorption in muscle as a function of depth at different frequencies.

**Figure 2.**
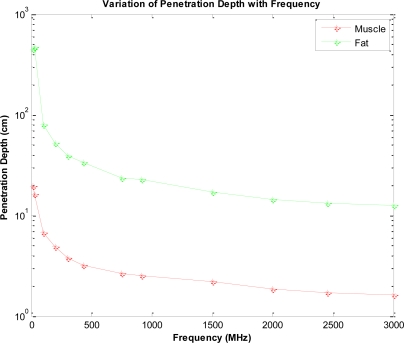
Variation of Penetration depth with frequency.

**Figure 3.**
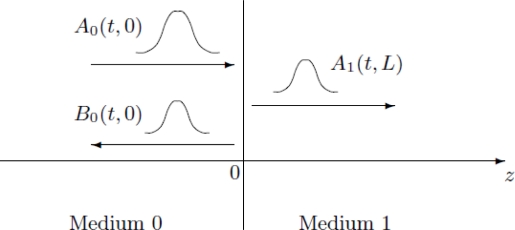
Scattering of a pulse by an interface.

**Figure 4.**
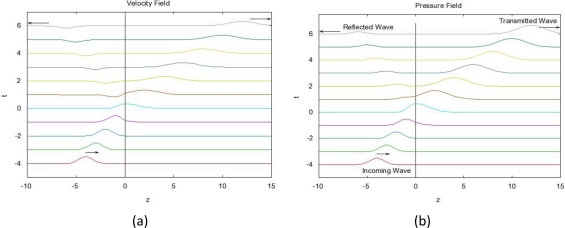
Scattering of a pulse by an interface separating two homogeneous half-spaces (*c*_0_, *ζ*_0_, *z* < 0) and (*c*_1_, *ζ*_1_, *z* > 0). Here the incoming right-going wave has a Gaussian profile, *c*_0_ = *ζ*_0_ = 1, and *c*_1_ = *ζ*_1_ = 2. The spatial profiles of the velocity field (a) and of the pressure field (b) are plotted at times *t* = −4, *t* = −3,..., *t* = 6.

**Figure 5.**
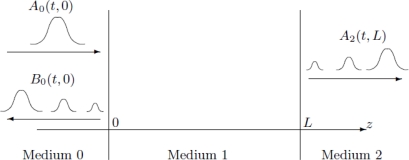
Scattering of a pulse by a single layer.

**Figure 6.**
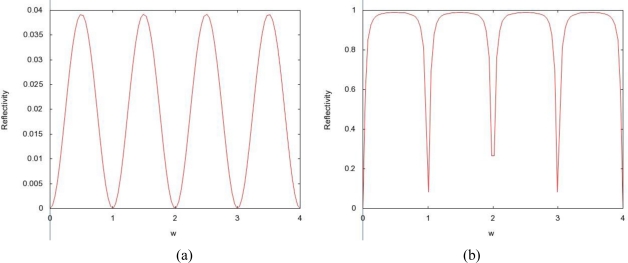
Reflectivity |𝒭̂(*ω*)|^2^ versus frequency for a single layer with *R*_0_ = −*R*_1_ = 0.1 (a) and *R*_0_ = −*R*_1_ = 0.9 (b). The period is *ω_c_* = *πc*_1_/*L*.

**Figure 7.**
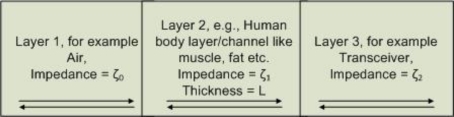
A propagation system consisting of 3 layers; air, human body channel, and a transceiver.

**Figure 8.**
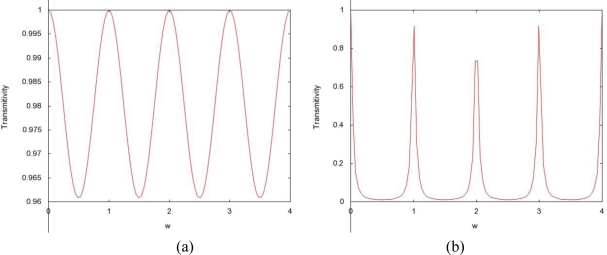
Transmitivity |𝒯̂(*ω*)|^2^ versus frequency for a single layer with *R*_0_ = −*R*_1_ = 0.1 (a) and *R*_0_ = −*R*_1_ = 0.9 (b). The period is *ω_c_* = *πc*_1_/2*L*.

**Figure 9.**
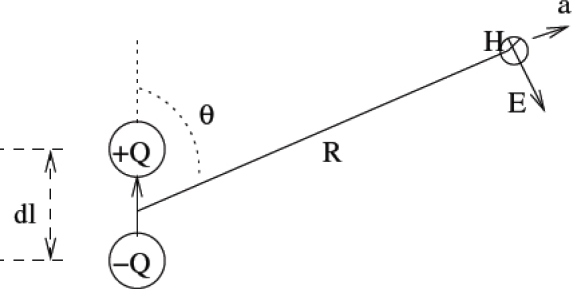
A Hertzian Dipole.

**Figure 10.**
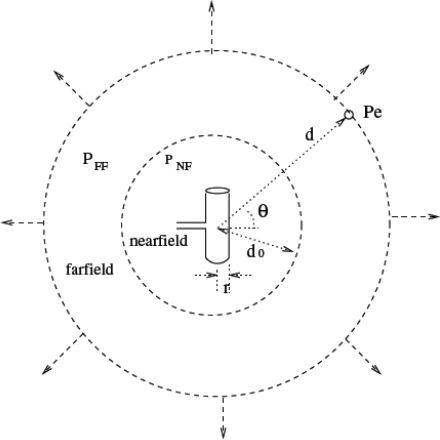
Field regions around a Hertzian Dipole.

**Figure 11.**
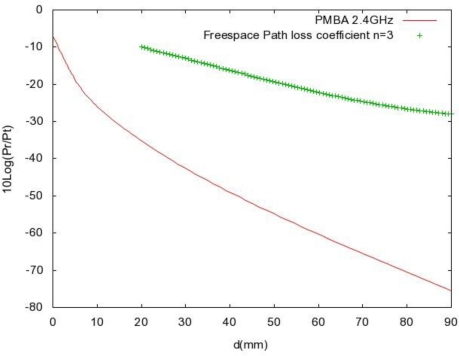
PMBA (tissue medium) and Freespace Pathloss at 2.4 GHz.

The permittivity of biological tissues depends on the type of tissues (e.g. skin, fat, or muscle), water content, temperature, and frequency. However, the permittivity and frequency may also determine how far the EM wave penetrates into the body. The term depth of penetration (D_p_) usually quantifies this. It is observed from Equations (1) and (2) that the wave gets attenuated as it propagates in the biological material along the z-axis. As shown in Figure 1, variation of radiation power density has been compiled for four different frequencies (27 MHz, 100 MHz, 433 MHz, and 1,500 MHz) with respect to the depth in muscle. At a given depth, usage of lower frequency results in a higher power density as illustrated in Figure 1. We discovered a distinct feature (demonstrated by Figure 2) which states that attenuation is more in fat than that in muscle with respect to frequency. Expressions for left- and right-going modes of a pulse have been derived in appendix B. Equations (19) and (20) express the reflectivity and transmitivity of a layer respectively. Equations (7) and (8) are the expressions of the left- and right-going modes respectively of a pulse when scattered by a single interface between two layers. Figure 4. shows the scattering of a Gaussian pulse by an interface separating two homogeneous half-spaces (*c*_0_, *ζ*_0_, *z* < 0) and (*c*_1_, *ζ*_1_, *z* > 0). The spatial profiles of the velocity field (a) and of the pressure field (b) are plotted at times *t* = −4, *t* = −3,..., *t* = 6. Reflectivity | 𝒭̂(*ω*)|^2^ versus frequency has been depicted by Figure 6. We can see here that, at a certain period *ω_c_* = *πc*_1_/*L*, the reflectivity maximum which is almost 0.04 for a layer with *R*_0_ = −*R*_1_ = 0.1 (a) and almost 1.0 for a layer with *R*_0_ = −*R*_1_ = 0.9 (b). In Figure 8, Transmitivity | 𝒯̂ (*ω*)|^2^ versus frequency curves have been drawn. Transmitivity has been found to vary periodically with a certain frequency. The period of this frequency band depends upon the choice of the layer thickness *L*. For a layer with *R*_0_ = −*R*_1_ = 0.1, transmitivity is about 1.0 (a) and for a layer with *R*_0_ = −*R*_1_ = 0.9, transmitivity is about 0.9 (b). Here the period is *ω_c_* = *πc*_1_/2*L*. From Equation (20), we can see that by the proper choice of the impedance of the layer to be $\zeta_{1}=\sqrt{\zeta_{0}\zeta_{2}}$ (so that *R*_0_ = *R*_1_) and the thickness *L* to be chosen so that *ωL*/(*πc*_1_) is half an integer (so that cos(2 *ωL*/*c*_1_) = −1), we can form a fully transmitting layer. Usually the thickness is chosen to be equal to a quarter of the wavelength, meaning *ωL*/(*πc*_1_) = ½. Therefore, from the results shown above, we can infer that a layer can either fully reflect or fully transmit any incoming wave at a certain frequency or frequency band. We can use these results to UWB by proper choice of impedance and the thickness L. Power loss in near filed and far field due to absorption has also been analyzed. A propagation loss model (PMBA) for homogeneous tissue bodies has been presented, which compares PMBA with the freespace propagation model ( $P_{R}=P_{T}G_{t}G_{r}{\left(\frac{\lambda }{4\pi d}\right)}^{n}$; with loss coefficient *n* = 3). A frequency range of (900 MHz to 3 GHz) has been considered here. We have been able to make a conclusion that, compared to freespace, there is an additional 30–35 dB of attenuation at small distances the far field (Figure 11). This loss increases further with the distance and frequency. It is argued that the human body cannot be considered as layered a media. However, we have been able to homogenize the human body channel, which is shown in Appendix C.

## 4. Conclusions

Employing UWB in WBAN involves a lot of promise, just as there are a number of relevant challenges. We studied the technical feasibility in this regard with a concentration in electromagnetic propagation of the signal across human body. Unlike conventional wireless channels, human body comes with a great deal of structural complexity requiring significantly different design considerations. The reflection and transmission coefficients of human body are heavily dependent upon the dielectric constants as well as upon the frequency. In this work, we investigated a layer-wise model for electromagnetic propagation across the components of the body in regard to such key aspects as scattering, reflectivity, and transmitivity. Naturally, the segmentation in precise layers are not what we come across in a body. But, the approximate model employing homogenization could help assess the aggregate behavior of the wireless communication involving implant devices, thus guiding the potential design issues for antenna characteristics, for instance. We also presented numerical depictions of some of the pertinent signal characteristics. From here on, one could expect to further improve the model in terms of suitable layering and other parameters of approximations.

## Appendix A

Let us assume that a biological medium is infinite in extent, source-free, isotropic, and homogeneous. The medium is isotropic if **ɛ** is a scalar constant, so ***D*** (electric displacement field vector) and ***E*** (electric field vector) are the same in every direction. A homogeneous medium is one for which *ε* (relative permittivity), *μ* (relative permeability), and *σ* (conductivity) are constants. For this case, Maxwell’s equations become:

(A.1) $$\vec{\nabla }\times \vec{E}=-\frac{\partial \vec{B}}{\partial t}$$

(A.2) $$\vec{\nabla }\times \vec{H}=\vec{J}+\frac{\partial \vec{D}}{\partial t}$$

(A.3) $$\vec{\nabla }\cdot \vec{B}=0$$

(A.4) $$\vec{\nabla }\cdot \vec{D}=0$$

Using following identities:

(A.5) $$\vec{\nabla }\times \vec{\nabla }\times \vec{E}=\vec{\nabla }\left(\vec{\nabla }\cdot \vec{E}\right)-\nabla^{2}\vec{E}$$

(A.6) $$\vec{B}=\mu \vec{H}$$

(A.7) $$\vec{D}=\varepsilon \vec{E}$$

(A.8) $$\vec{J}=\sigma \vec{E}$$

where, ε, μ, and σ are relative permittivity, relative permeability, and conductivity of the medium, **H** is magnetic field vector, **B** is magnetic flux density, **J** is the displacement current. We can find the expressions for *wave equation*:

(A.9) $$\left(\nabla^{2}-\mu \sigma \frac{\partial }{\partial t}-\mu \varepsilon \frac{\partial^{2}}{{\partial t}^{2}}\right)\left(\begin{array}{c} \vec{E}\\ \vec{H} \end{array}\right)=\left(\begin{array}{c} 0\\ 0 \end{array}\right)$$

In view of the fact that equations governing ***E*** and ***H*** in the biological material (Maxwell’s equations) are linear and keeping in mind that any arbitrarily time-varying function can be expressed as a sum of number of sinusoidal functions, time dependence of the fields, ***E*** and ***H***, can be given by the factor *e^jωt^* so that:

$$\begin{array}{l} \frac{\partial }{\partial t}\equiv j\omega \\ \frac{\partial^{2}}{\partial t^{2}}\equiv -\omega^{2} \end{array}$$

Using the relationships in (A.9), the wave equation becomes:

(A.10) $$\nabla^{2}\vec{E}+\gamma^{2}\vec{E}=0$$

where:

(A.11) $$\begin{array}{l} \gamma^{2}=\omega^{2}\mu \varepsilon -j\omega \mu \sigma \\ =\omega^{2}\mu \varepsilon_{0}\left(\varepsilon^{\prime }-j\frac{\sigma }{\omega \varepsilon }\right)\\ =\frac{\omega^{2}}{c^{2}}\left(\varepsilon^{\prime }-j\varepsilon^{\prime \prime }\right) \end{array}$$

And where *c* is the free space velocity (3 × 10^8^ m/s) and γ is the propagation constant. This is in general, a complex quantity and may be written in the form:

γ=α+jβ

where the attenuation constant is:

(A.12) $$\alpha =\frac{\sqrt{2}c}{\omega \sqrt{\varepsilon^{\prime }}{\left(\sqrt{1+{\left(\frac{\varepsilon^{\prime \prime }}{\varepsilon^{\prime }}\right)}^{2}}+1\right)}^{1/2}}$$

for $\frac{\varepsilon^{\prime \prime }}{\varepsilon^{\prime }}\leq 1$

(A.13) $$\alpha =\frac{\omega \sqrt{\mu \varepsilon }}{\sqrt{2}}\left(\frac{\varepsilon^{\prime \prime }}{\varepsilon^{\prime }}\right)$$

and the phase constant in radians per meter is:

(A.14) $$\beta =\frac{\sqrt{2}c}{\omega \sqrt{\varepsilon^{\prime }}{\left(\sqrt{1+{\left(\frac{\varepsilon^{\prime \prime }}{\varepsilon^{\prime }}\right)}^{2}}-1\right)}^{1/2}}$$

for $\frac{\varepsilon^{\prime \prime }}{\varepsilon^{\prime }}\leq 1$

(A.15) $$\beta =\omega \sqrt{\mu \varepsilon }\left[1+0.125{\left(\frac{\varepsilon^{\prime \prime }}{\varepsilon^{\prime }}\right)}^{2}\right]$$

Using Equation (A.15), the wavelength λ can be determined by:

(A.16) $$\lambda =\frac{2\pi }{\beta }$$

## Appendix B

In this appendix, we use a number of essential transformations of the wave equation that are specific to layered media. We consider the particular case in which the parameters of the medium vary in a piecewise-constant manner; in other words, we consider a stack of layers made of homogeneous media. We study the propagation of a normally incident plane wave, which enables us to reduce the problem to the one-dimensional acoustic wave equations [9]. We will see that the problem can be recast as a product of matrices corresponding to the scattering of the wave by the successive interfaces between the layers. This is a classical setup for waves propagating in this particular type of layered media, and it is extremely useful for direct numerical simulations. The equations for the three-dimensional velocity **u** and pressure *p* are:

(B.1) $$\rho \frac{\partial u}{\partial t}+\nabla p=0$$

(B.2) $$\frac{1}{K}\frac{\partial p}{\partial t}+\nabla \cdot u=0$$

where *ρ* is the density of the medium and *K* the bulk modulus of the medium. These two equations correspond respectively to conservation of momentum and mass. The density and bulk modulus are assumed to be spatially varying along the *z*-coordinate. If the initial conditions correspond to a plane wave that is normally incident to the layered medium, then the solution of the equations remains independent of the transverse variables, and the transverse velocity is zero. The system can then be reduced to the one-dimensional wave equations. It should be noted that more general conditions, corresponding in particular to point source, require a more general three-dimensional framework. In this appendix, we focus our attention to the one-dimensional case.

In one-dimensional medium the equations for the velocity *u* and pressure *p* are:

(B.3) $$\begin{array}{c} \rho (z)\frac{\partial u(t,z)}{\partial t}+\frac{\partial p(t,z)}{\partial z}=0\\ \frac{1}{K(z)}\frac{\partial p(t,z)}{\partial t}+\frac{\partial u(t,z)}{\partial z}=0 \end{array}\}$$

with *ρ* being the density and *K* the modulus of the medium, which are both functions of the spatial coordinate *z*. We write this system of equations in matrix form:

$$\frac{\partial }{\partial z}\left[\begin{array}{c} p(t,z)\\ u(t,z) \end{array}\right]=-\left[\begin{array}{cc} 0 & \rho (z)\\ K{\left(z\right)}^{-1} & 0 \end{array}\right]\frac{\partial }{\partial t}\left[\begin{array}{c} p(t,z)\\ u(t,z) \end{array}\right]$$

A diagonalization of the 2 × 2 matrix gives:

$$\left[\begin{array}{cc} 0 & \rho (z)\\ K{\left(z\right)}^{-1} & 0 \end{array}\right]=M{\left(z\right)}^{-1}\left[\begin{array}{cc} c{\left(z\right)}^{-1} & 0\\ 0 & -c{\left(z\right)}^{-1} \end{array}\right]M(z)$$

where:

$$M(z)=\left[\begin{array}{cc} \zeta {\left(z\right)}^{-1/2} & \zeta {\left(z\right)}^{1/2}\\ -\zeta {\left(z\right)}^{-1/2} & \zeta {\left(z\right)}^{1/2} \end{array}\right],\;\;M{\left(z\right)}^{-1}=\frac{1}{2}\left[\begin{array}{cc} \zeta {\left(z\right)}^{1/2} & -\zeta {\left(z\right)}^{1/2}\\ \zeta {\left(z\right)}^{-1/2} & \zeta {\left(z\right)}^{-1/2} \end{array}\right]$$

with $c(z)=\sqrt{K(z)/\rho (z)}$ and $\zeta (z)=\sqrt{K(z)\rho (z)}$ being respectively the local speed of sound and impedance. The system can then be written as:

$$\frac{\partial }{\partial z}\left[\begin{array}{c} p(t,z)\\ u(t,z) \end{array}\right]=-\frac{1}{c(z)}M{\left(z\right)}^{-1}\left[\begin{array}{cc} 1 & 0\\ 0 & -1 \end{array}\right]M(z)\frac{\partial }{\partial t}\left[\begin{array}{c} p(t,z)\\ u(t,z) \end{array}\right]$$

In this representation the material parameters *ρ* and *K* may either vary or remain constant (special case) with respect to the space coordinate *z*.

### Right- and Left-Going Waves

We consider the special case with a homogeneous medium in which the coefficients *ρ* and *K* are constant. Consequently the speed of sound *c* and the impedance *ζ* are constant, and the system can be written:

$$\frac{\partial }{\partial z}\left(M\left[\begin{array}{c} p(t,z)\\ u(t,z) \end{array}\right]\right)=-\frac{1}{c}\left[\begin{array}{cc} 1 & 0\\ 0 & -1 \end{array}\right]\frac{\partial }{\partial t}\left(M\left[\begin{array}{c} p(t,z)\\ u(t,z) \end{array}\right]\right)$$

Then if we define:

(B.4) $$\left[\begin{array}{c} A(t,z)\\ B(t,z) \end{array}\right]=M\left[\begin{array}{c} p(t,z)\\ u(t,z) \end{array}\right]=\left[\begin{array}{c} \zeta^{-1/2}p(t,z)+\zeta^{1/2}u(t,z)\\ -\zeta^{-1/2}p(t,z)+\zeta^{1/2}u(t,z) \end{array}\right]$$

it follows that:

(B.5) $$\frac{\partial }{\partial z}\left[\begin{array}{c} A(t,z)\\ B(t,z) \end{array}\right]=-\frac{1}{c}\left[\begin{array}{cc} 1 & 0\\ 0 & -1 \end{array}\right]\frac{\partial }{\partial t}\left[\begin{array}{c} A(t,z)\\ B(t,z) \end{array}\right]$$

The equations for A and B decouple:

$$\begin{array}{l} \frac{\partial A(t,z)}{\partial z}+\frac{1}{c}\frac{\partial A(t,z)}{\partial t}=0\\ \frac{\partial B(t,z)}{\partial z}-\frac{1}{c}\frac{\partial B(t,z)}{\partial t}=0 \end{array}$$

and the waves can be written *A* (*t*, *z*) = *a*(*t* − *z/c*) and *B* (*t*, *z*) = *b*(*t* + *z/c*) for some wave-shape functions *a* and *b*. Thus, in the constant medium case we have decomposed the wave into the right- and left-going waves A and B, which do not interact.

To fully specify the problem we have to prescribe initial conditions, for instance the velocity and pressure profiles at time *t =* 0:

$$u(t=0,z)=u_{0}(z),p(t=0,z)=p_{0}(z)$$

We then translate these initial conditions for *u* and *p* into initial conditions for the modes A and B:

$$\begin{array}{l} A_{0}(-z):=A(t=0,z)=\zeta^{-1/2}p_{0}(z)+\zeta^{1/2}u_{0}(z)\\ B_{0}(-z):=B(t=0,z)=-\zeta^{-1/2}p_{0}(z)+\zeta^{1/2}u_{0}(z) \end{array}$$

which gives the expressions for the modes:

$$A(t,z)=A_{0}(\mathrm{ct}-z),\;B(t,z)=B_{0}(\mathrm{ct}+z)$$

and finally the expressions for the wave:

$$\begin{array}{l} p(t,z)=\zeta^{1/2}\frac{A(t,z)-B(t,z)}{2}\\ u(t,z)=\zeta^{-1/2}\frac{A(t,z)+B(t,z)}{2} \end{array}$$

The initial conditions determine the mode decomposition and can be chosen to generate a pure right-going wave (if *p*_0_ ≡ *ζu*_0_) or a pure left-going wave (if *p*_0_ ≡ −*ζu*_0_).

A more physical way to generate a wave is to assume that the wave vanishes as *t* → −∞ and to introduce a source term in the acoustic wave equations:

$$\begin{array}{c} \rho \frac{\partial u(t,z)}{\partial t}+\frac{\partial p(t,z)}{\partial z}=F(t,z)\\ K^{-1}\frac{\partial p(t,z)}{\partial t}+\frac{\partial u(t,z)}{\partial z}=0 \end{array}$$

By assuming a point source *F* (*t*, *z*) = *ζ*^1/2^ *f*(*t*)*δ*(*z*) the system for A and B becomes:

$$\begin{array}{l} \frac{\partial A(t,z)}{\partial z}+\frac{1}{c}\frac{\partial A(t,z)}{\partial t}=\delta (z)f(t)\\ \frac{\partial B(t,z)}{\partial z}-\frac{1}{c}\frac{\partial B(t,z)}{\partial t}=-\delta (z)f(t) \end{array}$$

whose solutions are:

$$\begin{array}{l} A(t,z)=\{\begin{array}{c} f(t-z/c)\;\mathrm{if}\;z>0\\ 0\;\mathrm{if}\;z<0 \end{array}\\ B(t,z)=\{\begin{array}{c} 0\;\mathrm{if}\;z>0\\ f(t+z/c)\;\mathrm{if}\;z<0 \end{array} \end{array}$$

As a result, the velocity and pressure fields are:

$$\begin{array}{l} u(t,z)=\frac{\zeta^{-1/2}}{2}\{\begin{array}{c} f(t-z/c)\;\mathrm{if}\;z>0\\ f(t+z/c)\;\mathrm{if}\;z<0 \end{array}\\ p(t,z)=\frac{\zeta^{1/2}}{2}\{\begin{array}{c} f(t-z/c)\;\mathrm{if}\;z>0\\ -f(t+z/c)\;\mathrm{if}\;z<0 \end{array} \end{array}$$

This means that the source term generates two waves with equal energy that propagates to the right and to the left.

## Appendix C

### Homogenization:

For a channel like the human body where muscle, fat, blood cannot be considered as slabs or layers, we consider the idealized situation in which the parameters vary only with depth, and moreover, we make the important assumption that the variations are on a relatively fine scale. We assume that the scale of variation is small compared to the distance traveled by the pulse, as well as compared to the wavelength of the pulse. One may then expect that the waves are not strongly affected by the impedance in any particular layer. When a pulse propagates through such fine layers, the interaction with each layer is small, and propagation is not much affected. The pulse therefore travels as if the medium were homogeneous with the layers replaced by “averaged” ones. In general, we refer to this homogeneous medium as the homogenized medium. It is also referred to as an effective, average, or equivalent medium. We start by writing the medium parameters in the form *ρ* = *ρ*(*z/l*), *K* = *K*(*z/l*), with *l* a parameter that can be viewed as the layer size. Thus, ρ(z) is the variable density when observed through a magnifying glass with magnification factor 1/*l*. We then observe the fluctuations on their natural or intrinsic scale of variation. Typically, we will model ρ(z) as a stationary random process. Again we consider that the slab has constant parameters *ρ* ≡ *ρ̄* and *K* ≡ *K̄*, then $\bar{\zeta }=\sqrt{\bar{K}\bar{\rho }}$ and $\bar{c}=\sqrt{\bar{K}/\bar{\rho }}$. Assuming that the local propagation speed c varies with *z* but the impedance is constant. We choose the value *ζ̄* to be this constant. With these assumptions we have:

$$\frac{\rho (z)}{\bar{\rho }}=\frac{\bar{K}}{K(z)}=\frac{\bar{c}}{c(z)}$$

We introduce propagator *P_ω_* for each frequency ω, then we have:

(C.1) $$\frac{d}{\mathrm{dz}}P_{\omega }\;(0,z)=H_{\omega }\;(z,z/l)P_{\omega }\;(0,z),\;P_{\omega }\;(0,0)=I$$

where we have denoted the identity matrix by **I** and we have introduced:

$$\begin{array}{c} H_{\omega }\;(z,z^{\prime })=\frac{i\omega }{\bar{c}}\left[\begin{array}{cc} \left(\Delta^{\left(+\right)}(z^{\prime })-1\right) & \Delta^{\left(-\right)}(z^{\prime })e^{-2i\omega z/\bar{c}}\\ -\Delta^{\left(-\right)}(z^{\prime })e^{+2i\omega z/\bar{c}} & \left(1-\Delta^{\left(+\right)}(z^{\prime })\right) \end{array}\right]\\ \Delta^{\left(\pm \right)}(z)=\frac{1}{2}\left(\frac{\rho (z)}{\bar{\rho }}\pm \frac{\bar{K}}{K(z)}\right)\\ \Delta_{l}^{\left(\pm \right)}(z)=\Delta^{\left(\pm \right)}(z/l),z^{\prime }=\frac{z}{l} \end{array}$$

The matrix *P_ω_* (0, *z*) “propagates” the wave components from *z* = 0 to any other location *z* > 0, since the linearity of:

(C.2) $$\frac{d}{\mathrm{dz}}\left[\begin{array}{c} \hat{a}\\ \hat{b} \end{array}\right]=\frac{i\omega }{\bar{c}}\left[\begin{array}{cc} \left(\Delta_{l}^{\left(+\right)}-1\right) & \Delta_{l}^{\left(-\right)}e^{-2i\omega z/\bar{c}}\\ -\Delta_{l}^{\left(-\right)}e^{+2i\omega z/\bar{c}} & \left(1-\Delta_{l}^{\left(+\right)}\right) \end{array}\right]\;\left[\begin{array}{c} \hat{a}\\ \hat{b} \end{array}\right],0<z<L$$

implies that:

(C.3) $$\left[\begin{array}{c} \hat{a}(\omega ,z)\\ \hat{b}(\omega ,z) \end{array}\right]=P_{\omega }\;(0,z)\;\left[\begin{array}{c} \hat{a}(\omega ,0)\\ \hat{b}(\omega ,0) \end{array}\right]$$

for any *z* ∈ [0, *L*]. Equation (C.2) can be found by Fourier transforming *A*(*t*, 0) + *R*_0_*B*(*t*, 0) = *T*_0_ *f*(*t*), *T*_1_*A*(*t*, *L*) − *B*(*t*, *L*) = 0 with boundary conditions *â*(*ω*, 0) + *R*_0_*b̂*(*ω*, 0) = *T*_0_*f̂*(*ω*), $R_{1}e^{-2i\frac{\omega L}{\bar{c}}}\hat{a}(\omega ,L)-\hat{b}(\omega ,L)=0$, where *f̂*(*ω*) = ∫ *f*(*s*)*e^iωs^ ds*.

Equation (C.1) is diagonal and can be integrated by exponentiation:

(C.4) $$P_{\omega }(0,z)=\left[\begin{array}{cc} e^{i\omega S_{l}(z)} & 0\\ 0 & e^{-i\omega S_{l}(z)} \end{array}\right]$$

(C.5) $$S_{l}(z)=\int_{0}^{z}\left(\frac{1}{c(y/l)}-\frac{1}{\bar{c}}\right)\;\mathrm{dy}$$

To determine the effective medium that emerges in the limit of fine layering *l* → 0, we see from (C.5) that we need to study the behavior of $\int_{0}^{z}\left(\frac{1}{c(y/l)}-\frac{1}{\bar{c}}\right)\;\mathrm{dy}$ as *l* → 0. Homogenization can be illustrated using the simple model in which the medium is made up of independent and identically distributed layers of equal width *l* → 0. The medium is defined by one sequence of independent and identically distributed positive random variables C_n_ that are bounded and bounded away from zero. The local speed of propagation is given by:

$$c(z/l)=C_{\left[z/l\right]}$$

where [*x*] denotes the integer part of x. Since [*z/l*] → ∞ as *l* → 0, we can apply the law of large numbers to obtain:

(C.6) $$\begin{array}{ll} & \int_{0}^{z}\left(\frac{1}{c\left(\frac{y}{l}\right)}-\frac{1}{\bar{c}}\right)\;\mathrm{dy}=l\int_{0}^{\frac{z}{l}}c^{-1}(\tilde{y})\;d\tilde{y}\\ = & \underset{z}{\underset{︸}{l\;[z/l]}}\times \underset{𝔼\left[\frac{1}{C_{1}}\right]}{\underset{︸}{\frac{1}{\left[z/l\right]}\left(\sum_{j=0}^{[z/l]-1}\frac{1}{C_{j}}\right)}}+l\left(\frac{z}{l}-[z/l]\right)\frac{1}{C_{\left[z/l\right]}}\underset{\to }{l\to 0}\;z𝔼\left[\frac{1}{C_{1}}\right] \end{array}$$

The convergence is in the almost sure sense, for almost all realizations of the medium, or with probability one with respect to the randomness. In this setting, homogenization in the frequency domain means that we should choose *c̄* such that in the limit that *l* → 0 the propagator *P_ω_* (0, *z*) becomes the identity for all z. Using (C.4–C.5), we see that we must have:

$$\bar{c}={\left(𝔼\left[\frac{1}{C_{1}}\right]\right)}^{-1}$$

Thus, the harmonic mean of local propagation speeds is the homogenized or effective propagation speed. This effective propagation speed is frequency independent in this example, and therefore it is also the effective propagation speed in the time domain. We can get the transmitted and reflected waves in the Fourier domain:

$$\begin{array}{l} \hat{a_{1}}(\omega ,\;L)=\frac{T_{0}T_{1}}{1+R_{0}R_{1}e^{2i\frac{\omega L}{\bar{c}}}}\hat{f}(\omega )\\ \hat{b_{0}}(\omega ,\;0)=\frac{R_{0}+R_{1}e^{2i\frac{\omega L}{\bar{c}}}}{1+R_{0}R_{1}e^{2i\frac{\omega L}{\bar{c}}}}\hat{f}(\omega ) \end{array}$$
